# The Influence of Crops on the Content of Polycyclic Aromatic Hydrocarbons in Soil Fertilized with Manure and Mineral Fertilizers

**DOI:** 10.3390/ijerph192013627

**Published:** 2022-10-20

**Authors:** Ewa Mackiewicz-Walec, Sławomir Józef Krzebietke, Stanisław Sienkiewicz

**Affiliations:** 1 Department of Agrotechnology and Agribusiness, Faculty of Agriculture and Forestry, University of Warmia and Mazury in Olsztyn, 10-719 Olsztyn, Poland; 2 Department of Agricultural and Environmental Chemistry, Faculty of Agriculture and Forestry, University of Warmia and Mazury in Olsztyn, 10-719 Olsztyn, Poland

**Keywords:** light PAHs, heavy PAHs, total 16 PAHs, sugar beets, spring barley, maize, spring wheat, mineral fertilization, manure

## Abstract

Polycyclic aromatic hydrocarbons (PAHs) are mainly accumulated in soil. Plants secrete enzymes that transform or biodegrade PAHs in soil. Some plant species are more effective in stimulating the biodegradation of these pollutants than other species. This study was undertaken to evaluate the influence of crop rotation on PAH concentrations in soil. Four crops were grown in rotation: sugar beets, spring barley, maize, and spring wheat. Soil samples for the study were obtained from a long-term field experiment established in 1986 in Bałcyny, Poland. The concentrations of PAHs were analyzed in soil samples gathered over a period of 12 years (1998–2009). An attempt was made to evaluate the effect of crop rotation (sugar beets, spring barley, maize, and spring wheat) on PAH concentrations in soil. The content of PAHs in soil samples was measured by gas chromatography with flame ionization detection. Data were processed statistically by repeated measures ANOVA. The concentrations of ∑16 PAHs were lowest in soil after sugar beet cultivation, and highest in soil after maize cultivation. It can be concluded that maize was the plant with the greatest adverse effect on the content of heavy PAH in the soil, a completely different effect can be attributed to spring wheat, which has always been shown to reduce the content of heavy PAH in the soil. Weather conditions affected PAHs levels in soil, and PAH content was highest in soil samples collected in a year with the driest growing season. This arrangement suggests a greater influence of weather conditions than of the cultivated plant.

## 1. Introduction

Soil is an important aspect of the ecosystem that plays a crucial role in human population sustainability. Due to natural and anthropogenic activity, soil pollution has become a major environmental issue around the world [1]. Soil quality and soil fertility depletion decline have been threatening the ecological and economic sustainability of crop production [2].

PAHs are ubiquitous and highly mobile compounds that pose a serious environmental threat [3,4,5]. These compounds are the by-products of numerous chemical reactions, and they are formed when organic compounds are incompletely combusted during chemical processes. PAHs belong to persistent organic pollutants (POPs) found in the natural environment [6]. Due to their ubiquitous occurrence, recalcitrance, bioaccumulation potential, and carcinogenic activity, PAHs are a significant environmental concern [7,8,9].

PAHs present in the human environment are derived mainly from anthropogenic sources, whereas PAHs derived from natural sources are regarded as background PAHs. Naturally synthesized PAHs have fewer aromatic rings than PAHs from anthropogenic sources [10,11]. The local sources of pollution are responsible for higher PAH levels in soil [12]. PAHs are present in all components of the natural environment, including soil, water, and air. Numerous studies have shown that soil is the main source of exposure to PAHs [13] because 89.9% of these pollutants are accumulated in the soil environment. PAHs present in soil contaminate vegetables and crops, and they can be transferred to the food chain [14,15,16,17,18,19]. Liao [20] also reported that the uptake of organic pollutants by plants is an important part of the assessment of risks from crops grown on contaminated soils.

Plants’ roots absorb a range of natural and anthropogenic toxic compounds for which they have developed some extraordinary detoxification mechanisms. The phytoremediation of PAHs refers to the use of plants and associated soil microorganisms, in terms of reducing the concentrations or toxic effects of these contaminants in the environment [21]. Plants can absorb PAHs from various aspects of the environment, such as the atmosphere, water, and soil [22]. Phytoremediation comprises simple processes that differ on the ability of the plants to remove, immobilize, or degrade contaminants [9]. The mechanisms underlying phytoremediation are not well-understood, which has been a major obstacle in applying and practicing phytoremediation in large areas [23].

The aim of this study was to evaluate the influence of sugar beets, spring barley, maize, and spring wheat grown in three rotations on PAH concentrations in soil. During the long-term field experiments, different fertilization doses of plants with manure and mineral fertilizers were included.

## 2. Materials and Methods

### 2.1. Study Site and Experimental Design

The experimental material consisted of soil samples collected between 1998 and 2009 during a long-term field experiment established in Bałcyny near Ostróda (N: 53° 35′ 38.1″, E: 19° 50′56.1″). The soil in the study site was classified as Haplic Luvisol developed from sandy loam. Soil composition was determined before the experiment, and 1 kg of soil contained 100.0 mg K, 53.2 mg Mg, 41.3 mg P, 7.9 g of organic carbon, and 0.79 g of total nitrogen. The analyzed soil samples were slightly acidic and (pH in 1 M KCl = 6.2).

The analysis involved soil samples from treatments fertilized with manure and mineral fertilizers, as well as treatments supplied with mineral fertilizers only (Table 1). The amounts of nutrients supplied to plants were comparable in both systems. Four crops were grown: sugar beets, spring barley, maize, and spring wheat. After spring wheat harvest (treatment No. 8), soil was limed with 2.5 t CaO ha^−1^ every four years. Manure was applied at 40 t ha^−1^ every two years before sugar beet and maize cultivation. Spring barley and spring wheat were grown in the second year after manure application. The chemical composition and content of heavy metals and PAHs (total PAHs, light PAHs, and heavy PAHs) in manure were described previously by Krzebietke et al. [24].

Between 1998–2009, all agronomic practices were applied in accordance with specific crop requirements (Table S1). Phenological observations were conducted during plant growth, and the main developmental stages are described in Table S2. At the end of the growing season, soil samples were collected from the arable layer (0–30 cm) in each plot with the use of Egner’s sampler. The samples were air dried and passed through a sieve with 2 mm mesh size.

The content of 16 PAHs as priority pollutants by the US EPA (naphthalene, acenaphthene, acenaphthylene, fluorene, phenanthrene, anthracene, fluoranthene, pyrene, benzo(a)anthracene, chrysene, benzo(b)fluoranthene, benzo(k)fluoranthene, benzo(a)pyrene, indeno(1,2,3-cd)pyrene, dibenzo(a,h)anthracene, and benzo(g,h,i)perylene)–Restek Corporation (SV Calibration Mix#5—Cat. No. 31011)—was determined with the Trace GC Ultra ITQ900 gas chromatography system equipped with a TRIPlus autosampler (Thermo Fisher Scientific, Waltham, MA, USA) and a flame ionization detector (FID).

The total content of 16 PAHs was determined by the method previously described by Krzebietke et al. [13]. The total content of heavy PAHs (benzo(a)anthracene, benzo(a)pyrene, benzo(b)fluoranthene, benzo(k)fluoranthene, benzo(g,h,i)perylene, indeno(1,2,3-cd)pyrene, and dibenzo(a,h)anthracene) and the total content of light PAHs (naphthalene, acenaphthene, acenaphthylene, fluorene, anthracene, phenanthrene, fluoranthene, pyrene, and chrysene) were determined in soil samples.

### 2.2. Statistical Analysis

In each group, data were tested for normal distribution before statistical analysis. The equal variance hypothesis was validated with Mauchly’s sphericity test. Data that did not satisfy the sphericity condition were analyzed with the use of Wilk’s lambda test and Pillai’s trace criterion. The Shapiro–Wilk test revealed that data did not have normal distribution; therefore, they were log transformed. Data were processed in Tukey’s post hoc HSD test at *p* < 0.05. Statistical analyses were performed in the Statistica program [25].

### 2.3. Weather Conditions

Air temperature and precipitation levels varied across years during the long-term field experiment (1998–2009) (Table 2). Mean annual temperatures were higher in 2000 (8.8 °C) and 2008 (8.6 °C) and lower in 1998 (7.3 °C) and 2004 (7.4 °C). Mean annual temperatures in 2005 (7.7 °C) and 2009 (7.9 °C) most closely approximated the long-term average (1998–2009) (8.0 °C). During the growing season, the highest temperatures were noted in 2002 (15.2 °C), 2006 (15.1 °C), and 2009 (14.8 °C), whereas the lowest temperatures were observed in 1998 (13.8 °C), 2001 (13.9 °C), and 2004 (13.4 °C). Precipitation levels were lowest in 2005 (501.3 mm) and highest in 2007 (767 mm).

In years of sweet beet cultivation (2002 and 2006), mean annual temperatures exceeded the long-term average by 0.9 °C and 0.8 °C, respectively. During spring barley (2003) and spring wheat (2005) cultivation, mean annual temperatures were similar to the long-term average (14.4 °C and 14.3 °C, respectively). In 2004, the mean annual temperature during the growing season (13.4 °C) was not optimal for maize production.

Precipitation levels varied considerably during the 12-year field experiment. The highest total precipitation and the highest precipitation during the growing season were noted in 2006 and 2007. Precipitation was particularly high in 2007, when total rainfall between January and December exceeded the long-term average by 22%, and total rainfall in the growing season (April to September) exceeded the long-term average by 26%. In turn, 2005 was a particularly dry year, when total precipitation did not exceed 510 mm, and precipitation in the growing season did not exceed 270 mm.

## 3. Results

### 3.1. Total Content of Light PAHs

**Figure 1:**
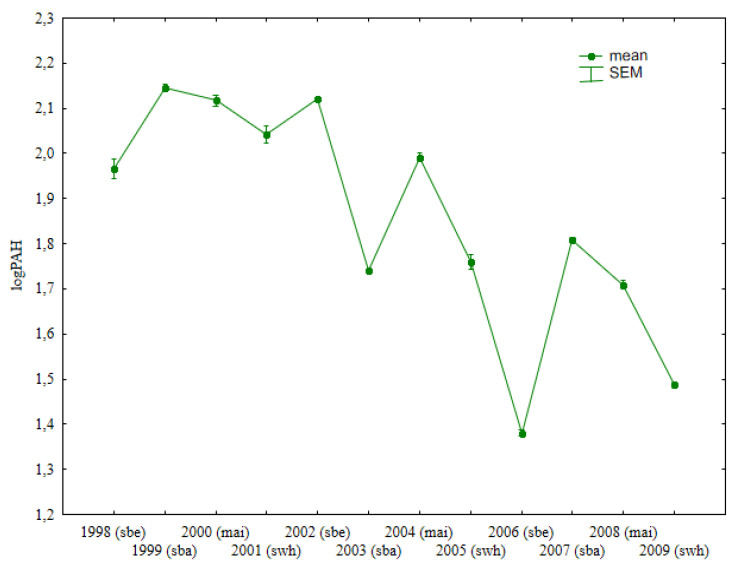
Total content of light PAHs in soil under different crops (log transformed data for 1998–2009).

The total content of light PAHs (naphthalene, acenaphthene, acenaphthylene, fluorene, anthracene, phenanthrene, fluoranthene, pyrene, and chrysene) was lowest (29.5 µg kg^−1^) in soil samples collected after sweet beet harvest in 2006 (Figure 1, Table S3). This parameter was lower in soil sampled after spring wheat cultivation. In each year of spring wheat cultivation (2001, 2005, 2009), the total concentrations of light PAHs were lower than in soil sampled after the harvest of the preceding crops. The analyzed parameter was higher in all years of spring barley cultivation. Both spring barley and spring wheat were grown in the second year after manure application.

**Figure 2:**
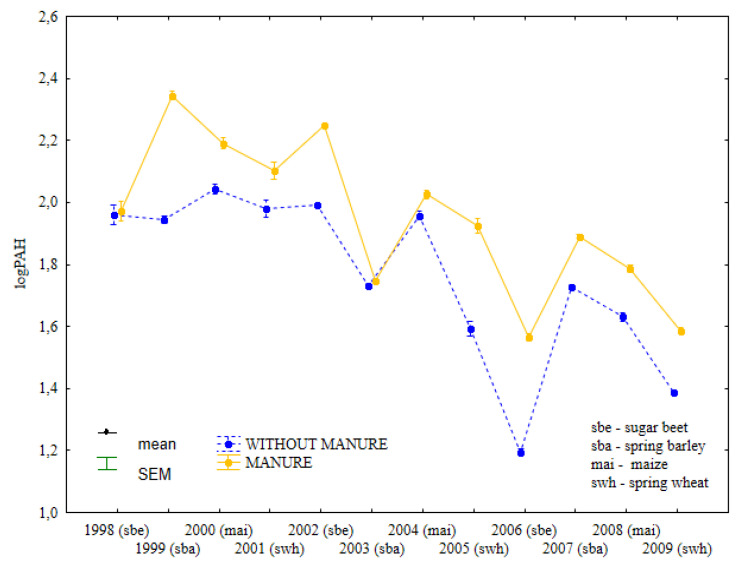
Total content of light PAHs in soil under different crops in treatments supplied with both manure and mineral fertilizers or mineral fertilizers only (log transformed data for 1998–2009).

During the entire experiment, the total content of light PAHs was higher in soil fertilized with manure than in soil supplied with mineral fertilizers only (Figure 2, Table S3). In 1998 and 2004, the concentrations of light PAHs were similar in treatments that were and were not fertilized with manure. From 1998–2002, the content of light PAHs remained relatively stable in soil supplied with mineral fertilizers only. In the same period, treatments supplied with manure and mineral fertilizers differed considerably in the levels of light PAHs.

From 2003–2009, similar changes in the content of light PAHs were observed in soil fertilized with manure and mineral fertilizers and in treatments supplied with mineral fertilizers only. The concentrations of light PAHs increased significantly in treatments that were regularly fertilized with manure after spring barley cultivation (1999) and sweet beet cultivation (2002), whereas the content of light PAHs did not change in soil supplied with mineral fertilizers only. Combined manure and mineral fertilization, and mineral fertilization only exerted opposite effects on the total content of light PAHs in soil samples collected after maize cultivation in 2000.

### 3.2. Total Content of Heavy PAHs

**Figure 3:**
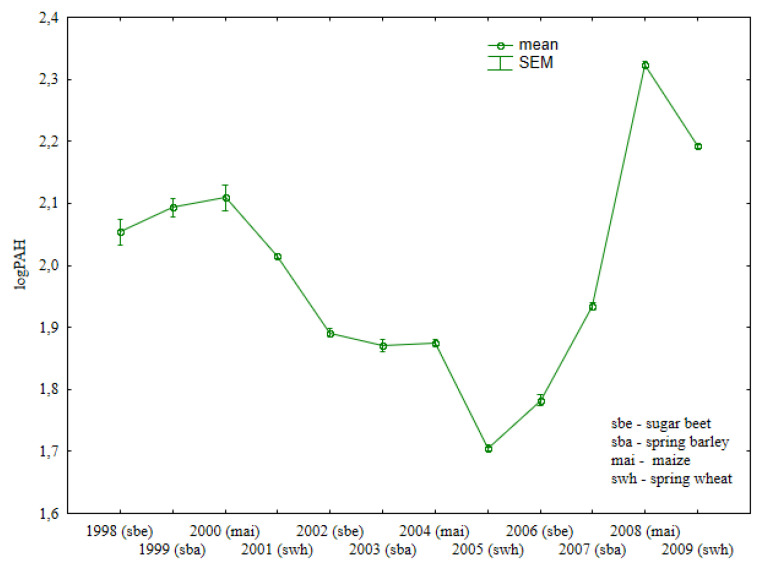
Total content of heavy PAHs in soil under different crops (log transformed data for 1998–2009).

Maize was least effective in reducing the concentrations of heavy PAHs in soil (Figure 3, Table S3). In contrast, spring wheat grown after maize decreased heavy PAH levels in soil in all years of cultivation. Sweet beets exerted a less adverse effect on the content of heavy PAHs in soil than maize.

The content of the analyzed substances was similar in soil after spring barley (1999) and maize (2000) cultivation. Similar observations were made after the harvest of sugar beets, spring barley, and maize in 2002, 2003, and 2004.

**Figure 4:**
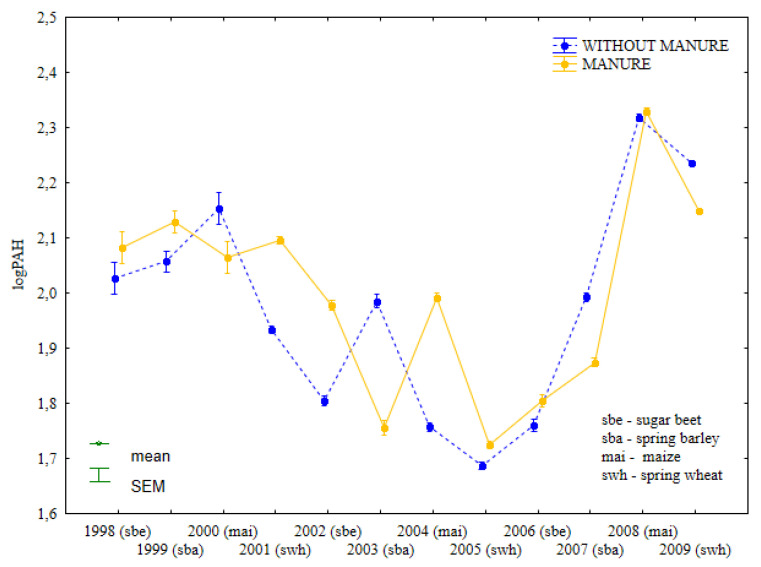
Total content of heavy PAHs in soil under different crops in treatments supplied with both manure and mineral fertilizers or mineral fertilizers only (log transformed data for 1998–2009).

The concentrations of heavy PAHs in soil differed significantly in treatments fertilized with manure and mineral fertilizers, and in treatments supplied with mineral fertilizers only (Figure 4, Table S4). In most cases, the content of heavy PAHs was higher in soil fertilized with manure than in treatments supplied with mineral fertilizers alone. However, heavy PAH levels were only higher in treatments supplied with mineral fertilizers than in treatments fertilized with both manure and mineral fertilizers in 2002 (after maize cultivation), 2003, and 2007 (after spring barley cultivation). The extent to which specific crops affect the concentrations of heavy PAHs in soil is difficult to determine.

The concentrations of the studied substances were similar in soil supplied with mineral fertilizers after sugar beet harvest in 2002 and in treatments that were regularly fertilized with manure in 2006. After maize cultivation in 2008, the total content of heavy PAHs was similar in treatments supplied with both manure and mineral fertilizers and in treatments supplied with mineral fertilizers only.

### 3.3. Total Content of 16 PAHs

**Figure 5:**
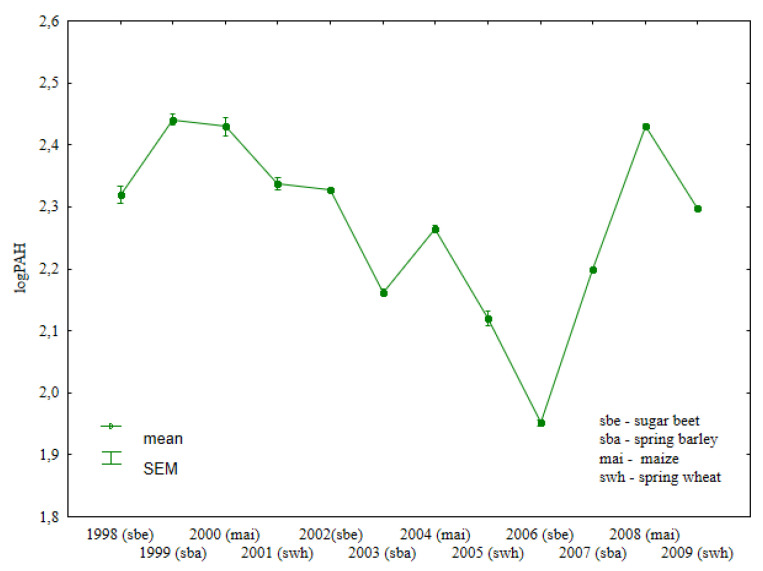
Total content of 16 PAHs in soil under different crops (log transformed data for 1998–2009).

The total content of 16 PAHs in soil was lowest (102.4 µg kg^−1^) after sugar beet harvest in 2006 (Figure 5, Table S3). Oleszczuk and Baran [26] also reported lower PAH concentrations in the rhizosphere of beetroots than in control soil. In the present experiment, 2006 was one of the years with the highest mean temperature (15.1 °C) and the highest precipitation (475 mm) during the growing season (Table 2), which could have contributed to the decrease in the total content of 16 PAHs in soil. According to Eriksson et al. [27], low temperature and aerobic conditions considerably inhibit PAH biodegradation in soil. Wang et al. [28] found that PAH concentrations in the arable layer decreased with a rise in annual precipitation. In the current study, total PAH content was highest (282.3 µg kg soil) in a year with the driest growing season (2008). In turn, Wyszkowski and Ziółkowska [29] demonstrated that maize could contribute to a reduction in PAH levels in soil. The content of 16 PAHs in soil was low after spring wheat cultivation (2001, 2005, 2009). Spring barley did not decrease the soil levels of 16 PAH. The content of the analyzed substances in soil increased in 1998 and 2008 relative to the preceding years, whereas the opposite was noted in 2003.

The concentrations of the analyzed substances in soil were similar after sugar beet cultivation in 1998 and 2002, and similar observations were made after maize harvest in 2000 and 2008 (Table S3). The content of PAHs in soil sampled after spring barley and spring wheat harvest differed significantly across years. These observations suggest that temperature and precipitation considerably affect PAH levels in soil.

**Figure 6:**
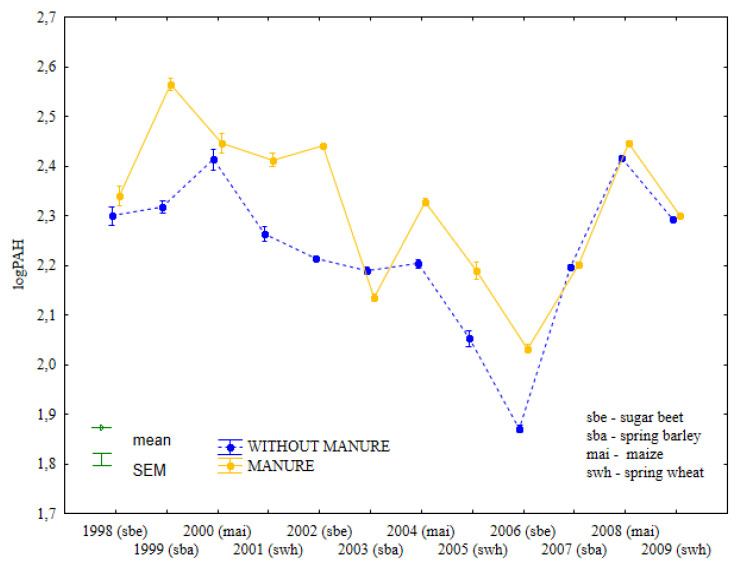
Total content of 16 PAHs in soil under different crops in treatments supplied with both manure and mineral fertilizers or mineral fertilizers only (log transformed data for 1998–2009).

The total content of 16 PAHs in soil varied considerably across years, which suggests that weather conditions exert a greater influence on the studied parameter than crop species. In most cases, PAH concentrations were significantly higher in treatments fertilized with both manure and mineral fertilizers than in treatments supplied with mineral fertilizers only, although an inverse relationship was noted in 2003 (Figure 6, Table S3). The same crop species exerted different effects on PAH levels in treatments that were and were not fertilized with manure. In soil regularly fertilized with manure, the content of 16 PAHs increased significantly after sugar beet cultivation in 2002 relative to the preceding year, whereas an inverse relationship was noted in the treatment supplied with mineral fertilizers only. In turn, after maize cultivation in 2000, the total concentrations of 16 PAHs increased relative to the previous year in the treatment supplied with mineral fertilizers only but decreased in the treatment fertilized with manure.

The total content of 16 PAHs in soil regularly fertilized with manure was identical after maize cultivation in 2000 and 2008. The same observation was made after the harvest of spring barley in 2003 and 2007.

## 4. Discussion

Global problems with the environment and increasing pollution have made environmental issues a major priority. Human beings have realized that environmental investments are needed to maintain better world conditions for future generations [30]. Soil pollution is one of the most serious environmental problems globally due to the weak self-purification ability, long degradation time, and high cost of cleaning soil pollution [31]. PAHs are widespread in the environment and pose a serious threat to the soil ecosystem [32].

Soil is a porous and complex mixture, containing inorganic elements and compounds formed and anthropogenic pollutants [33]. Numerous factors can influence the rate and extent of PAH biodegradation in soil [34,35,36,37,38,39,40,41], including organic matter content, nutrient content, fertilization, C:N:P ratio, microbial counts, electron acceptors, temperature, moisture, pH, and oxygen concentration. PAHs are removed from soil both with [42] and without microbial involvement [43]. They usually undergo two-stage degradation. At first, PAH levels decrease rapidly, but their degradation is slowed down over time [39].

Diverse plants remediate different pollutants at different rates through one or multiple mechanisms. Plants can metabolize and destroy contaminants within plant tissues through the process known as phytodegradation; when degradation of contaminants occurs in the rhizosphere, the process is called rhizodegradation. Phytoremediation is an environmentally friendly technique that utilizes plants to immobilize, uptake, reduce toxicity, stabilize, or degrade the compounds that are released into the environment from different sources [44]. Phytoremediation is, therefore, one of the soil remediation technologies with the greatest potential as a result of their unique advantages, including low cost, lack of secondary pollution, and large-area application [7,45].

Rhizodegradation, namely the degradation of pollutants in the root zone, relies on the interactions between plants, soil, and soil-dwelling microorganisms [46]. PAHs are biodegraded more effectively in the rhizosphere under direct influence of plant roots [47]. The biodegradation of PAHs is one of the major mechanisms for their removal from environment. However, unlike microorganisms, the degradation pathways of organic pollutants in plant systems are not completely clear [48]. Nitrogen promotes the growth of plants that remove pollutants from soil (phytoremediation), and PAHs are more effectively degraded in soils that are abundant in nitrogen [49]. Kołwzan et al. [50] demonstrated that microbial counts were higher in the rhizosphere than in soil without roots. Both microorganisms and plants secrete enzymes that act as surface active agents and decrease organic pollutant levels [51]. Root secretions, such as sugars, vitamins, and amino acids, contribute to an increase in microbial counts.

Rhizosphere microorganisms protect plants against stress caused by excessive pollution (by synthesizing protective compounds) and pathogens, and they degrade contaminants [52]. Plants significantly contribute to PAH removal from soil by modifying microbial abundance and the composition of microbial communities [53,54,55]. Root secretions stimulate the development of microorganisms, thus, affecting the rate of pollutant degradation [56]. According to Gramss et al. [57], Krauss et al. [58], and Rentz et al. [59], the phytoremediation of soils contaminated with PAHs involves direct PAH uptake by plants, plant secretions and enzymes that degrade PAHs in the rhizosphere, and increased abundance of soil-dwelling microorganisms that decompose PAHs. Plant-based methods, such as rhizodegradation, are very promising for the remediation of petroleum-contaminated soils [60].

Crop species can exert varied effects on different types of soil-dwelling bacteria, and root secretions can accelerate the biodegradation of pollutants [61,62]. Root secretions contain chemical compounds that are characteristic of a given plant species and influence soil microbial communities [63]. According to Marchal et al. [64] and Fu et al. [65], active rhizosphere bacterial communities speed up PAH decomposition in soil.

Plant species differ in their ability to stimulate the biodegradation of PAHs [66]. The rhizosphere is characterized by both synergistic and antagonistic relationships between microorganisms, roots, and environmental conditions [67]. According to Oleszczuk and Baran [61], root secretions affect the soil microflora and contribute to a decrease PAH levels. Fluorene, anthracene, and pyrene are more effectively removed in the rhizosphere of sunflowers, compared with the rhizosphere of other crop species, such as wheat, oats, and maize. In the work of Liste and Alexander [68], phenanthrene and pyrene concentrations were higher in the rhizosphere of tall fescue grass and wheat than in unplanted soil. Cheema et al. [18] reported that tall fescue grass effectively reduced phenanthrene and pyrene levels in soil. Fu et al. [65] analyzed the content of 15 PAHs in soil and found that perennial ryegrass decreased the concentrations of anthracene (by 13%), fluorene (by 18%), and phenanthrene (by 18%) after 7 months of cultivation, in comparison with unplanted soil. In a study by White et al. [69], plants and fertilization clearly decreased the content of persistent PAHs. According to Lin [70], maize roots and tops of plants directly accumulate PAHs from aqueous solutions and from air in proportion to exposure levels. In the work by Odika [71], the mean PAHs concentration levels of the maize types were very low, compared to the permissible limit established by European Food Safety. Houshani et al. [72] demonstrated that that pyrene had a negative effect on the growth variables of maize plants. According to Houshani [48], the maize plant had potential for the degradation of phenanthrene and pyrene. The study suggests that the use of the maize plant with surfactant is an alternative technology for remediation of PAHs-contaminated soils [20]. Additionally, [60] found that maize can be used to accelerate the rhizodegradation of petroleum hydrocarbons in soil.

## 5. Conclusions

Maize was least effective in removing heavy PAHs from soil, whereas spring wheat decreased the content of heavy PAHs in all years and treatments. Soil contamination with PAHs of 282.3 µg kg^−1^ was highest in a year with the driest growing season, which suggests that weather conditions exert a greater influence on PAH levels in soil than crop species. The results of this study indicate that weather conditions contribute to considerable seasonal variations in the PAH concentrations in soil. Future studies of long-term fertilization experiment should be carried out to confirm present results and to prove the lack of negative impact of optimal long-term fertilization of plants in rotation on the accumulation of toxic compounds from the PAHs in the soils.

## Figures and Tables

**Table 1:** Mineral fertilization plan.

No	Variant	SUGAR BEET *Spring Barley* *Maize* Spring Wheat *			
		N	P	K	Mg
		Dose [kg ha^−1^]			
1	N_0_P_0_K_0_	0 *0* **0** 0 *	0 * **0 **	0 0 **0** 0 *	0 0 * **0**
2	N_1_P_1_K_1_	60 *30* **60** 40 *	34.9 * **26.2**	66.4 *33.2 ***49.8** 24.9 *	0 0 * **0**
3	N_2_P_1_K_1_	120 *60* **120** 80 *	34.9 * **26.2**	66.4 *33.2* **49.8** 24.9 *	0 0 * **0**
4	N_3_P_1_K_1_	180 *90* **180** 120 *	34.9 * **26.2**	66.4 *33.2* **49.8** 24.9 *	0 0 * **0**
5	N_2_P_1_K_2_	120 *60* **120** 80 *	34.9 * **26.2**	132.8 *66.4* **99.7** 49.8 *	0 0 * **0**
6	N_2_P_1_K_3_	120 *60* **120** 80 *	34.9 * **26.2**	199.3 *99.7* **149.7** 74.7 *	0 0 * **0**
7	N_2_P_1_K_2_Mg	120 *60* **120** 80 *	34.9 * **26.2**	132.8 *66.4* **99.7** 49.8 *	48.2 *18.1* * **24.1**
8	N_2_P_1_K_2_MgCa	120 *60* **120** 80 *	34.9 * **26.2**	132.8 *66.4* **99.7** 49.8 *	48.2 *18.1* * **24.1**

**Table 2:** Mean monthly temperature and total precipitation in the study site.

Month	Year													
	1998	1999	2000	2001	2002	2003	2004	2005	2006	2007	2008	2009	1998–2009	2015
Temperature (°C)														
I	0.4	−0.5	−2.1	−1.2	−1.1	−3.8	−6.9	0.6	−8.7	2.4	0.7	−3.7	−2.0	0.6
II	2.4	−2.0	1.4	−1.4	2.8	−5.2	−1.1	−3.2	−3.4	−2.0	2.3	−1.5	−0.9	0.3
III	0.5	3.8	2.2	0.8	3.5	1.4	2.4	−1.4	−2.5	5.4	2.9	1.9	1.7	4.6
IV	9.0	8.3	10.9	7.3	7.3	6.1	7.7	7.7	7.8	7.3	7.7	9.7	8.1	7.2
V	13.3	11.1	13.5	12.2	16.1	14.2	11.0	12.5	12.5	13.7	12.3	12.2	12.9	12.1
VI	16.2	16.7	15.9	13.8	15.9	16.5	14.5	14.9	16.0	17.5	16.5	14.7	15.8	15.7
VII	16.3	19.1	15.3	19.5	19.3	18.9	16.2	18.9	21.0	17.5	18.3	18.9	18.3	18.0
VIII	15.1	16.9	16.9	18.4	19.8	17.3	18.2	16.8	17.3	18.2	17.7	18.5	17.6	21.3
IX	13.0	15.3	11.2	12.0	12.5	13.7	13.0	15.3	15.9	12.6	11.9	14.7	13.4	14.2
X	7.1	7.9	11.1	10.5	6.4	4.8	9.2	8.3	10.2	7.4	8.6	5.9	8.1	6.6
XI	−3.0	1.6	7.4	2.2	3.1	4.9	2.4	2.8	5.6	1.0	4.0	5.2	3.1	5.1
XII	−3.2	0.4	1.2	−4.6	−6.6	1.3	2.3	−1.1	4.2	0.4	−0.2	−1.7	−0.6	3.8
I−XII	7.3	8.2	8.8	7.5	8.3	7.5	7.4	7.7	8.0	8.4	8.6	7.9	8.0	9.1
IV−IX	13.8	14.6	14.0	13.9	15.2	14.4	13.4	14.3	15.1	14.5	14.1	14.8	14.3	14.7
Precipitation (mm)														
I	38.7	18.3	28.8	17.7	41.6	14.1	28.9	50.3	15.3	110.2	30.8	16.2	34.2	28.5
II	32.2	8.2	45.5	13.3	54.5	6.0	60.7	21.4	26.7	14.6	33.9	14.7	27.6	8.8
III	38.1	20.2	52.9	30.2	37.0	11.8	28.2	29.3	3.1	27.9	47.1	68.0	32.8	46.0
IV	44.5	101.6	20.2	43.5	10.0	23.6	51.5	22.0	24.2	26.8	33.8	3.7	33.8	23.4
V	58.3	69.1	32.5	3.3	90.1	78.6	87.1	68.2	93.2	79.7	48.4	89.6	68.8	25.4
VI	141.9	155.6	33.1	48.8	72.5	60.7	90.6	35.4	83.5	60.8	27.8	133.1	78.7	43.0
VII	57.5	75.5	104.2	135.1	43.2	118.2	78.8	83.9	27.1	176.5	47.0	82.2	85.8	71.0
VIII	58.3	53.0	140.9	81.8	87.3	34.9	89.3	39.6	141.7	81.0	103.1	25.7	78.1	13.0
IX	21.8	18.4	46.8	99.3	60.5	19.1	41.9	17.9	105.6	65.4	17.0	15.6	44.1	51.2
X	55.0	70.0	4.9	35.1	144.5	66.1	77.6	19.3	34.3	48.9	104.6	58.5	59.9	20.8
XI	37.9	50.1	53.3	47.5	28.2	39.4	27.8	31.1	107.3	50.0	40.5	40.8	46.2	80.8
XII	45.3	54.8	39.7	24.1	6.8	48.6	39.5	82.9	60.0	25.2	29.4	29.6	40.49	80.4
I−XII	629.5	694.8	602.8	607.7	676.2	521.1	701.9	501.3	722.0	767.0	563.4	577.7	630.5	492.3
IV−IX	382.3	473.2	377.7	439.8	363.6	335.1	439.2	267.0	475.3	490.2	277.1	349.9	389.2	227.0

## Data Availability

Not applicable.

## Supplementary Materials

The following supporting information can be downloaded at: https://www.mdpi.com/article/10.3390/ijerph192013627/s1, Table S1: Dates of agronomic practices in the cultivation of sugar beets, spring barley, maize, and spring wheat. Table S2: Phenological growth stages of sugar beets, spring barley, maize, and spring wheat. Table S3: Content of PAHs in soil during the long-term field experiment (1998–2009) across years (Y), and the interactions between years (Y) and organic fertilization (O).
