# Supplementary material for: The Influence of Crops on the Content of Polycyclic Aromatic Hydrocarbons in Soil Fertilized with Manure and Mineral Fertilizers
Source: Int J Environ Res Public Health. 2022 Oct 20;19(20):13627. doi: 10.3390/ijerph192013627 (PMC9602616; doi:10.3390/ijerph192013627)
Supplement: Supplementary file 1 [file ijerph-19-13627-s001.zip › ijerph-1973324-supplementary.pdf]

Table S1: Dates of agronomic practices in the cultivation of sugar beets, spring barley, maize, and spring wheat.

Agronomic practices	**Sugar beet** (1998, 2002, 2006)	Agronomic practices	**Spring barley** (1999, 2003,2007)	Agronomic practices	**Maize** (2000, 2004, 2008)	Agronomic practices	**Spring wheat** (2001, 2005, 2009)
Ca fertilization (2.5 t ha^{-1})	03/09-05/09	Fall plowing	23/10-25/10	Manure application (40 t ha^{-1}- experimental series with manure)	25/09-17/10	Fall plowing	14/10-30/10
Skimming, harrowing	04/09-08/09	Harrowing	20/03-08/04	Fall plowing	26/09-17/10	Cultivation unit, harrowing	27/03-11/04
Manure application (40 t ha^{-1}- experimental series with manure)	11/10-22/10	NPK fertilization in accordance with experimental design, cultivation unit, harrowing	26/03-15/04	Cultivation unit, harrowing	30/03-28/04	NPK fertilization in accordance with experimental design, cultivation unit, harrowing	06/04-29/04
Disking	13/10-15/10	Harrowing, Sowing, post-sowing harrowing	27/03-16/04	NPK fertilization in accordance with experimental design, cultivation unit, harrowing	26/04-29/04	Sowing	07/04-29/04
Fall plowing	24/10-28/10	Herbicide application: *Granstar* 18 kg ha^{-1}, *Chwastox Extra* 3.0 l ha^{-1}	14/05-28/05	Sowing	27/04-30/04	Herbicide application: *Granstar* 20 g ha^{-1} + *Trend* 0.05 l ha^{-1}	14/05-19/05
Cultivation unit, harrowing	01/04-20/04	Top-dressing, treatment 4 (N- 34% ammonium nitrate, in accordance with experimental design)	23/05-03/06	Herbicide application: *Milagro Extra 60SC* 0.6 l ha^{-1}	29/05-02/06	Top-dressing, treatment 4 (34% ammonium nitrate, in accordance with experimental design)	25/05-10/06
NPK fertilization in accordance with experimental design, cultivation unit, harrowing	08/04-22/04	Fungicide application: *Escudo Forte* 0.8 l ha^{-1}, *Juwel* 0.9 l ha^{-1}, Insecticide application: *Karate* 0.25 l ha^{-1} (cereal leaf beetle)	05/06-17/06	Top-dressing, treatment 8 in accordance with experimental design	25/05-01/07	Fungicide application: *Amistar* 1.0 l ha^{-1}, Insecticide application: *Decis* 0.25 l ha^{-1} (cereal leaf beetle),	05/06-29/06
Harrowing, Sowing	10/04-28/04	Insecticide application: *Decis* 0.25 l ha^{-1} (cereal leaf beetle), *Fastac* 0.1 l ha^{-1} (cereal leaf beetle), *Charisma* 1.5 l ha^{-1}	11/06-21/06	Harvest	22/09-04/10	Insecticide application: *Charisma* 1.5 l ha^{-1}, *Nurella* 0.6 l ha^{-1}	20/06-01/07
Herbicide application: *Pyramin* 3 kg ha^{-1}	10/04-12/04	Harvest with a plot combine harvester	02/08-09/08			Harvest with a plot combine harvester	19/08-29/08
Inter-row weeding	22/04-23/05						
Sugar beet thinning	17/05-08/06						
Top-dressing of sugar beets (treatment 4)	23/05-09/06						
Harvest	06/10-13/10						

Table S2: Phenological growth stages of sugar beets, spring barley, maize, and spring wheat.

Growth stage	Sugar beets (1998, 2002, 2006)	Growth stage	Spring barley (1999, 2003, 2007)	Growth stage	Maize (2000, 2004, 2008)	Growth stage	Spring wheat (2001, 2005, 2009)
BBCH 10	02/05-12/05	BBCH 10	13/04-28/04	BBCH 10	05/05-12/05	BBCH 10	17/04-30/04
BBCH 19	06/05-16/05	BBCH 19	17/04-02/05	BBCH 19	09/05-15/05	BBCH 19	20/04-05/05
BBCH 31	29/05-26/06	BBCH 21	28/04-09/05	BBCH 30	12/06-24/07	BBCH 21	05/05-13/05
BBCH 39	10/06-02/07	BBCH 29	01/05-14/05	BBCH 39	17/06-29/07	BBCH 29	09/05-17/05
		BBCH 30	11/05-23/05	BBCH 51	01/06-05/08	BBCH 30	19/05-25/05
		BBCH 39	16/05-29/05	BBCH 59	17/06-10/08	BBCH 39	22/05-30/05
		BBCH 51	02/06-13/06	BBCH 61	04/07-17/08	BBCH 51	15/06-29/06
		BBCH 59	06/06-16/06	BBCH 67	26/06-18/08	BBCH 58	22/06-07/07
		BBCH 61	07/06-23/06	BBCH 73	08/07-21/08	BBCH 61	19/06-18/07
		BBCH 73	24/06-10/07	BBCH 75	14/08-28/08	BBCH 65	30/06-22/07
		BBCH 75	28/06-15/07	BBCH 83	12/07-03/09	BBCH 73	25/06-26/07
		BBCH 83	11/07-27/07	BBCH 85	27/08-08/09	BBCH 75	29/06-29/07
		BBCH 85	14/07-30/07	BBCH 89	12/09-18/09	BBCH 83	22/07-02/08
		BBCH 87	19/07-04/08			BBCH 85	26/07-06/08
		BBCH 89	23/07-07/08			BBCH 87	29/07-10/08
						BBCH 89	02/08-14/08

*BBCH - Biologische Bundesanstalt, Bundessortenamt und CHemische Industrie coding.

Table S3: Content of PAHs in soil during the long-term field experiment (1998-2009) across years (Y), and the interactions between years (Y) and organic fertilization (O).

Total content of 16 PAHs in soil

Crop species	Sugar beets			Spring barley			Maize			Spring wheat		
**year**	**1998**	**2002**	**2006**	**1999**	**2003**	**2007**	**2000**	**2004**	**2008**	**2001**	**2005**	**2009**
manure - μg kg^{-1}	227.9	278.3	126.9	377.6	145.9	199.0	286.3	222.5	295.0	268.5	162.5	220.2
(log transformed data)	(2.340^{d})	(2.441^{b})	(2.032^{j})	(2.564^{a})	(2.135^{i})	(2.201^{h})	(2.446^{b})	(2.327^{de})	(2.446^{b})	(2.412^{c})	(2.189^{h})	(2.301^{ef})
without manure - μg kg^{-1}	205.0	167.6	77.9	215.6	168.1	185.2	277.1	172.2	269.6	193.7	118.8	210.6
(log transformed data)	(2.299^{ef})	(2.215^{h})	(1.871^{k})	(2.318^{e})	(2.189^{h})	(2.196^{h})	(2.413^{bc})	(2.203^{h})	(2.416^{bc})	(2.263^{g})	(2.052^{j})	(2.294^{f})
mean - μg kg^{-1}	216.5	223.0	102.4	296.6	157.0	192.1	281.7	197.3	282.3	231.1	140.7	215.4
(log transformed data)	(2.319^{b})	(2.328^{b})	(1.951^{h})	(2.441^{a})	(2.162^{f})	(2.199^{e})	(2.429^{a})	(2.265^{d})	(2.431^{a})	(2.337^{b})	(2.121^{g})	(2.297^{c})

Total content of light PAHs in soil

manure - μg kg^{-1}	98.0	182.2	42.0	233.4	75.5	99.3	166.3	120.5	71.4	132.4	95.5	53.2
(log transformed data)	(1.972^{fg})	(2.249^{b})	(1.565^{l})	(2.345^{a})	(1.746^{ij})	(1.891^{h})	(2.191^{c})	(2.026^{ef})	(1.786^{i})	(2.102^{d})	(1.925^{gh})	(1.586^{l})
without manure - μg kg^{-1}	94.4	100.3	17.0	98.6	65.2	68.4	125.3	109.6	50.9	101.4	59.2	25.5
(log transformed data)	(1.959^{g})	(1.993^{f})	(1.196^{n})	(1.945^{gh})	(1.732^{j})	(1.727^{j})	(2.044^{e})	(1.957^{g})	(1.631^{k})	(1.981^{fg})	(1.594^{kl})	(1.387^{m})
mean - μg kg^{-1}	96.2	141.2	29.5	166.0	70.3	83.9	145.8	115.0	61.1	116.9	77.4	39.3
(log transformed data)	(1.965^{d})	(2.121^{ab})	(1.380^{i})	(2.145^{a})	(1.739^{f})	(1.809^{e})	(2.117^{b})	(1.991^{d})	(1.709^{g})	(2.042^{c})	(1.760^{f})	(1.487^{h})

Total content of heavy PAHs in soil

manure - μg kg^{-1}	129.9	96.1	84.9	144.2	70.4	99.7	120.0	102.2	223.6	136.1	67.0	167.0
(log transformed data)	(2.082^{d})	(1.978^{f})	(1.805^{i})	(2.129^{cd})	(1.756^{j})	(1.874^{h})	(2.065^{d})	(1.992^{ef})	(2.329^{a})	(2.096^{cd})	(1.724^{jk})	(2.148^{c})
without manure - μg kg^{-1}	111.0	67.3	60.9	116.9	102.9	116.8	151.9	62.6	218.7	92.2	59.5	185.1
(log transformed data)	(2.026^{e})	(1.805^{i})	(1.761^{j})	(2.057^{de})	(1.986^{f})	(1.992^{ef})	(2.154^{c})	(1.757^{j})	(2.318^{a})	(1.934^{g})	(1.687^{k})	(2.236^{b})
mean - μg kg^{-1}	120.4	81.7	72.9	130.5	86.7	108.2	135.9	82.3	221.2	114.2	63.3	176.0
(log transformed data)	(2.054^{d})	(1.891^{g})	(1.783^{h})	(2.093^{c})	(1.871^{g})	(1.933^{f})	(2.109^{c})	(1.875^{g})	(2.323^{a})	(2.015^{e})	(1.705^{i})	(2.192^{b})

* log transformed data followed by different letters a,b,c (…) are significantly different at $p < 0.05$
